# Thermo-Electrochemical Cells Based on Carbon Nanotube Electrodes by Electrophoretic Deposition

**DOI:** 10.1007/s40820-016-0082-8

**Published:** 2016-01-29

**Authors:** Weijin Qian, Mingxuan Cao, Fei Xie, Changkun Dong

**Affiliations:** Institute of Mirco-Nano Structure & Optoelectronics, Wenzhou University, Wenzhou, 325035 People’s Republic of China

**Keywords:** Thermo-electrochemical cells, Carbon nanotubes, Electrophoretic deposition, Power conversion efficiency

## Abstract

Drawbacks of low efficiency and high cost of the electrode materials have restricted the wide applications of the thermo-electrochemical cells (TECs). Due to high specific areas and electrical conductivities, the low cost multi-walled carbon nanotubes (MWNTs) are promising alternative electrode materials. In this work, the MWNT films of up to 16 cm^2^ were synthesized on stainless steel substrates by the electrophoretic deposition (EPD) to make the thermo-electrochemical electrodes. MWNT electrodes based on TECs were characterized by cyclic voltammetry and the long-term stability tests with the potassium ferri/ferrocyanide electrolyte. The TECs reached the current density of 45.2 A m^−2^ and the maximum power density of 0.82 W m^−2^. The relative power conversion efficiency of the MWNT electrode is 50 % higher than that for the Pt electrode. Meanwhile, the TECs was operated continuously for 300 h without performance degradation. With the priorities of low cost and simple fabrication, EPD-based MWNT TECs may become commercially viable.

**Electronic supplementary material:**

The online version of this article (doi:10.1007/s40820-016-0082-8) contains supplementary material, which is available to authorized users.

## Introduction

Harvesting of low grade heat (<130 °C) is considered an effective sustainable energy source. Thermo-electrochemical cells (TECs) utilize the temperature-dependent electrochemical redox potentials to convert the thermal energy to electrical energy. Comparing with other thermal energy harvesting techniques, such as the thermoelectrics, thermocouples, and stirling engines [1–4], TECs have great potential for wide applications due to advantages of simple design, maintenance-free, environment-friendly, and low cost.

**Fig. 1:**
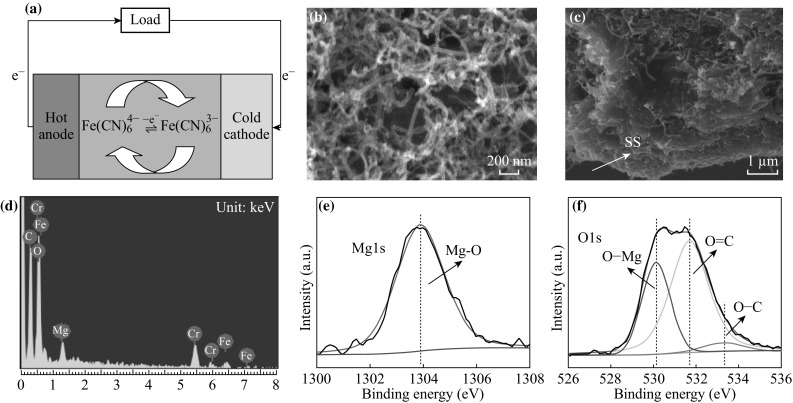
**a** Schematic of a TEC with nanostructured electrodes showing concentration gradients of the ferri/ferrocyanide redox ions during power generation. **b**, **c** SEM images of MWNT film on stainless steel substrate. **d** EDS analysis of the MWNT electrode. **e**, **f** XPS spectra of Mg 1s and O 1s for the MWNT sample

As shown in Fig. 1a, the two half cells of the TECs are held at different temperatures, causing a difference in the redox potential of the mediator around the anode and cathode [5]. Electrons are generated at the anode due to the oxidation reaction of ferrocyanide. When traveling through the cathode, electrons would be consumed from the reduction reaction of ferricyanide. The ingredient of the electrolyte keeps almost unchanged owing to the balance of oxidized and reduced species in the solution [5]. As a result, the current and output power can be acquired continuously.

The electrode exchange current density is one of the most important factors in energy conversion for TECs. In practice, the current of TECs can be described from the relation: *I* = *V*/*R*, where *V* represents the voltage between the two working electrodes, and the resistance *R* consists of four parts, i.e., charge transfer, ohmic, solution diffusion, and thermal diffusion resistances [6, 7]. To get high exchange current densities, the redox couples, such as the ferri/ferrocyanide electrolyte, are commonly selected in TECs [8, 9]. In the selection of electrode materials, the fast charge transfer property and low resistance at the electrode/electrolyte interface are important factors. Platinum is the conventional electrode material due to high surface catalytic activity for oxidation and reduction reactions. However, it is hard to promote Pt-based TECs in engineering fields due to high cost and low conversion efficiency [6, 8]. With the development of nanotechnology [10–13], carbon nanotubes (CNTs) have been employed in different electrochemical devices [14–18], such as lithium-ion batteries, supercapacitors, and fuel cells due to large specific surface area and high catalytic activity. Recently, applications of MWNTs in TECs are widely investigated [19–23]. In the preparation of the MWNT electrode, the chemical vapor deposition (CVD) growth is widely applied [8, 9, 22]. The MWNT TECs electrodes prepared by CVD show promising electrical contact and stability properties.

Electrophoretic deposition (EPD) is an effective technique to produce CNTs films with various advantages, including fewer requirements on the type & shape of the substrate, large-scale production capability, and low cost [24]. In this work, we prepared the TECs electrodes by EPD of MWNTs on metal substrates. The TECs presented excellent long-term operation stability and substantial higher energy conversion efficiency than that for Pt-based TECs. This investigation suggests that EPD method may be applicable for MWNTs-based TECs.

## Experimental

The MWNT material, with lengths from 10 to 30 μm, outer diameters of approximately 10 nm, and purity of >90 %, was purchased from XFNANO Materials. The MWNTs were first filtered and washed with acetone, then sonicated in concentrated nitric acid for 20 h. After the processing, carboxylic and other oxygen-containing groups were decorated on MWNTs surfaces [24]. During EPD, the carboxylic MWNTs were first dispersed in ethanol (0.1 g L^−1^) and sonicated for about 1 h. Then magnesium chloride powder material (MgCl_2_, Aladdin) was added into the suspension. Subsequently, the stainless steel substrate (SS) and counter electrode were immersed into the suspension with distance of 1 cm. Different substrates of surface areas from 0.5 to 16 cm^2^ were employed. After the deposition, the MWNT electrodes were annealed in vacuum at 750 °C.

The morphologies of the MWNT film were observed by scanning electron microscopy (SEM; JEOL SM-6700F). The compositions of the as-prepared products were characterized by energy-dispersive X-ray analysis (EDS), and X-ray photoelectron spectroscopy (XPS; PHI 5000 VersaProbe). The tensile tests of the samples were carried out by Instron 3343 instrument to investigate the adhesion between MWNT films and the substrates with the uncertainty of about 15 %. During the test, the MWNT-SS sample was fixed by a clamp, and the MWNT film was wrapped by the adhesive tape. The tape grabbing the MWNT film was pulled away until the film peeled off from the substrate.

The cyclic voltammetry (CV) measurements were conducted using a Zahner IM6 electrochemical workstation. The 3-electrode tests were conducted at room temperature with the Ag/AgCl saturated in KCl solution as the reference electrode and a platinum foil as the counter electrode. CVs were tested using 0.1 M K_4_Fe(CN)_6_ aqueous solution with 0.5 M NaCl as the supporting electrolyte at the rate of 5 mV S^−1^.

The characteristic performances of the MWNT-based TECs, including the open-circuit potential (*V*_oc_), the short-circuit current (*I*_sc_), and the output power, were investigated in I-shaped TECs and the stability was tested in the U-shaped TECs. The 0.4 M potassium ferrocyanide (K_4_Fe(CN)_6_·3H_2_O, Aladdin) and ferricyanide (K_3_Fe(CN)_6_, Aladdin) aqueous solution were employed as the electrolyte due to its high Seebeck coefficient [9]. For the I-shaped TECs, measurements were conducted in a glass tube with the internal diameter of 8 cm and the distance of two electrode of 5 cm. The hot side temperature was controlled by the resistive heating and the cold side was immerged in an ice water with the temperature difference of 40 °C. The electrode temperatures were measured by OMEGA thermocouple probes. The maximum power (*P*_max_) generated by the MWNT electrodes could be attained when the external load resistance is equal to the internal resistance. For the U-shaped TECs, the distance between the two electrodes was 7 cm with the temperature difference of 15 °C. The hot side temperature (40 °C) was controlled by a resistive heater, and the cold side temperature (25 °C) was controlled by a recirculation water chiller. The potentials and currents generated from TECs were monitored using the KEITHLEY 2440 sourcemeter.

## Results and Discussions

**Fig. 2:**
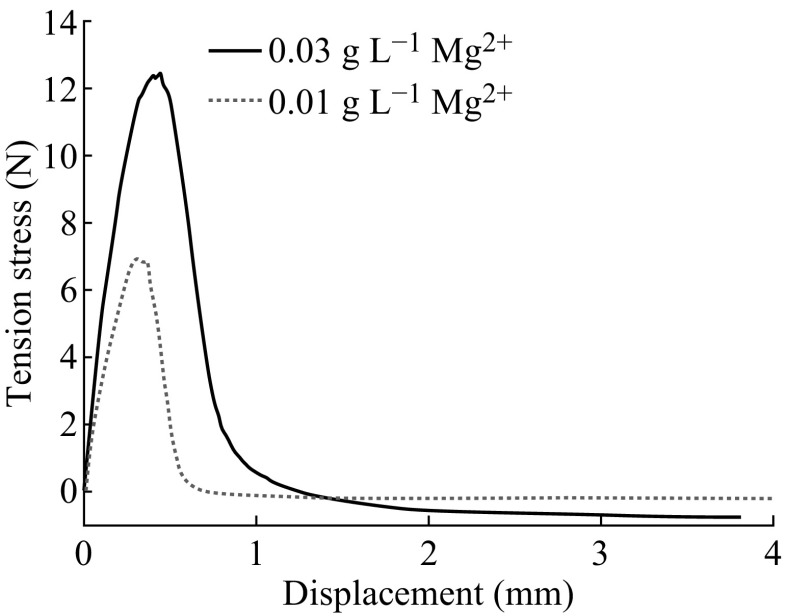
Tensile test of the MWNT film adhesion with the existence of MgO

Typical morphologies and composition characterizations of the MWNT samples are shown in Fig. 1. The top and side SEM images of the MWNT films are presented in Fig. 1b, c. As shown in Fig. 1b, the MWNTs are randomly oriented on the SS substrate with diameters of 30–50 nm. From the side SEM images (Fig. 1c), the MWNT film and Mg^2+^ form the pasting substance after the heat treatment. The EDS analysis result is shown in Fig. 1d. The signals of Fe and Cr are from the stainless steel substrate. The O signal mainly comes from the MgO due to the heat treatment of the products, while MgO could improve the adhesiveness between MWNTs and the substrate [25, 26], benefiting the efficiency and stability of TECs. XPS was applied to further investigate the composite of the film. As shown in Fig. 1e, the Mg 1 s peak at 1303.9 eV was detected, which was from the Mg–O bond and higher than the peak of metallic Mg (Mg1 s at ~1303 eV) [27]. For the O 1 s spectrum (Fig. 1f), three peaks at 530.1, 531.7, and 533.3 eV could be assigned to the O–Mg, O–C, and O=C bonds, respectively [28, 29]. It is worth noting that the peak at ~533.3 eV should not be assigned to the peroxide magnesium species because the products were conducted through the annealing process at 750 °C [27]. The XPS analysis confirmed the existence of MgO species. The tensile test was conducted to investigate the function of MgO on the adhesion between the MWNT film and the substrate, as shown in Fig. 2. The maximum stresses of 12.38 and 6.95 N correspond to Mg^2+^ contents of 0.03 and 0.01 g L^−1^, respectively, showing clearly adhesion enhancement with the existence of MgO. In addition, the lower concentration of Mg^2+^ (<0.03 g L^−1^) would bring CNTs to form the inhomogeneous film on the SS substrate, and the excessive Mg^2+^ would decrease the conductivity of the CNTs electrode due to the formation of MgO after the annealing treatment.

**Fig. 3:**
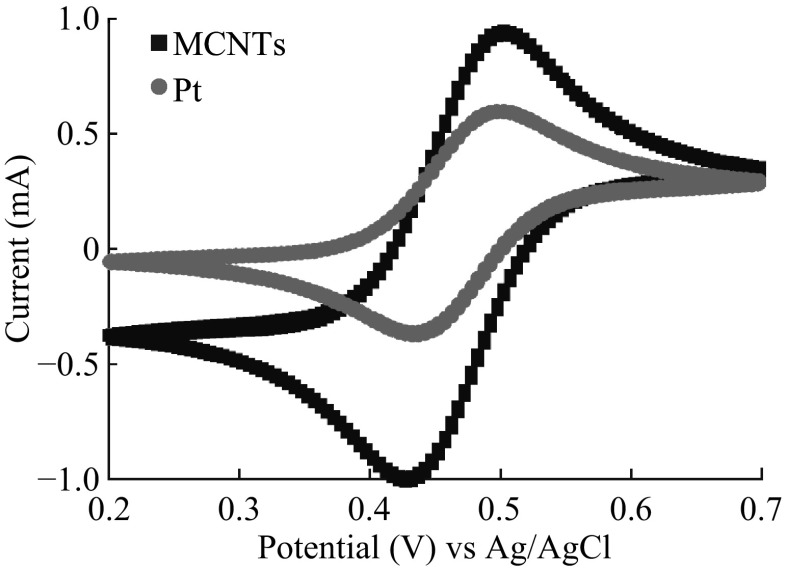
Cyclic voltammograms for platinum foil and MWNT/SS electrodes in 100 mM K_4_Fe(CN)_6_ solution. *Working electrode* MWNT/SS plate or Pt foil of 1 × 0.5 cm^2^, *counter electrode* Pt of 2.0 × 2.0 cm^2^, *reference electrode* Ag/AgCl in saturated KCl solution, *scan rate* 5 mV S^−1^

Cyclic voltammetry (CV) tests were conducted to investigate the electroactive surface area (ESA) of the electrode materials, as shown in Fig. 3. ESA can be described as

1 $$I_{P - f }\propto \sqrt {D\nu } C_{0} A$$

[30–32], and the Faradaic peak current *I*_p−f_ depends on the diffusion coefficient (*D*), initial concentration (*C*_o_), scan rate (*v*), and electroactive surface area (*A*). Generally, CV runs at low scan rates reflect the electrochemical equilibrium at electrode surfaces. The peak current of the MWNT electrode is larger than that of Pt with the same physical areas. Since both MWNT and the Pt show the reversible reactions, higher peak current for the MWNT electrode can be attributed to the increase of ESA. Under the scan rate of 5 mV S^−1^, the separations between the reduction and oxidation peaks for Pt and MWNT/SS electrodes are 65 and 72 mV, respectively, suggesting the similar reaction processes for both electrode materials. The charge transfer at the interface of the MWNT film and the electrolyte is as high as Pt electrode because of the fast kinetics [33], which is probably due to one-dimensional nanostructure, good crystallinity, and high localized electron density of states near the Fermi level with MWNT [8, 34].

**Fig. 4:**
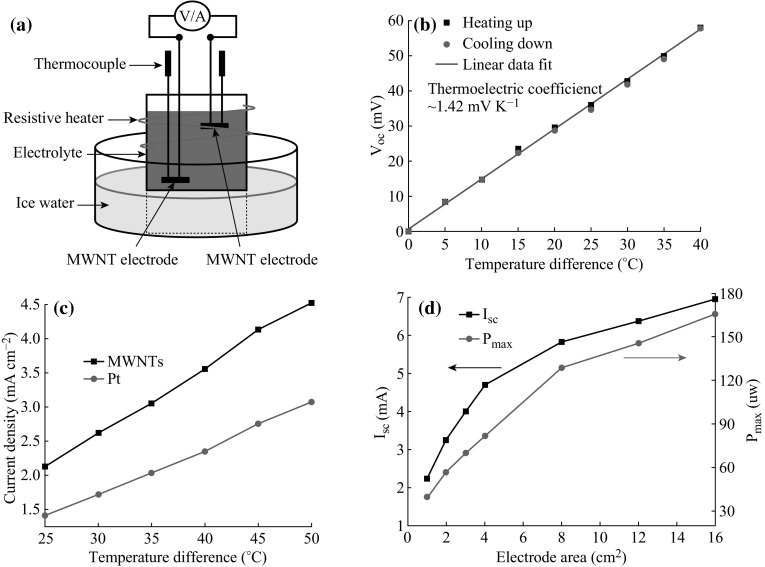
**a** Experimental setup for TEC performance measurements. **b** Thermoelectric coefficient measurements for 0.4 M ferro/ferricyanide (Fe(CN)_6_^4−^/Fe(CN)_6_^3−^) redox couple from the test of the dependence of open-circuit potential on the temperature difference between the hot and cold electrodes. The electrode area was 0.25 cm^2^ and the inter-electrode spacing was 4 cm. **c** *J*_SC_ versus temperature difference between electrodes. The distance between electrodes was 5 cm, and the electrode area was 0.4 cm^2^. **d** *I*_sc_ and *P*_max_ versus MWNT electrode areas. Temperature difference was 40 °C and the inter-electrode distance was 4 cm

Figure 4a shows the I-shaped TECs for performance investigations. The Seebeck coefficient can be described by the thermodynamics equation of the redox couple as follows: *S* = △*V*/△*T* = △*S*_B,A_/(*nf*), where *S* represents the Seebeck coefficient of the given redox couple, and △*T* are the electrode potential and the temperature difference between the two electrodes, respectively, △*S*_B,A_ is the reaction entropy of the redox reaction, *n* is the number of transferred electrons of the reaction, and *f* is Faraday’s constant. The Seebeck coefficient is constant for certain electrolyte, no matter what type of electrode Pt or MWNTs/SS is selected. *V*_oc_ (△*V*) is generated from the temperature difference between the two electrodes. As shown in Fig. 4b, *V*_oc_ and △*T* show linear relationship and the Seebeck coefficient was 1.42 mV K^−1^, in good agreement with previous reports [6, 8, 9].

Figure 4c shows that the current densities increased with the temperature differences, and *J*_SC_ of the MWNT electrode was about 50 % higher than that of the Pt electrode at the same temperature difference, contributed to good conductivity and high specific surface area for the MWNT film. Under the temperature difference of 50 °C, *J*_SC_ and *J*_SC_/Δ*T* reached 45.2 A m^−2^ and 0.91 A m^−2^ K^−1^ for MWNT electrodes, comparing with 30.5 A m^−2^ and 0.61 A m^−2^ K^−1^ for Pt electrodes.

To evaluate the performances of the MWNT-based TECs, the power conversion efficiencies *η*, expressed below, are calculated for both electrodes [22, 35, 36]:

2 $$\eta = \frac{{0.25V_{oc} I_{sc} }}{{AK\left( {\Delta T/d} \right)}}$$

3 $$\eta_{r} = \eta /\left( {\Delta T/\Delta_{h} } \right),$$

where *A* is the electrode front area, *k* is the thermal conductivity of the electrolyte, △*T* and *d* are the temperature difference and the distance between electrodes, respectively, *η*_r_ is the energy conversion efficiency compared to carnot efficiency, and *T*_h_ is the temperature of the hot side, measured from the front surfaces of the electrodes. The *η*_r_ for the MWNT electrode is 0.9, 50 % higher than that for the Pt electrode and similar with the results reported by Hu [8], which might be due to higher conductivity and lower thermal resistance at electrode/substrate junctions and better ESA of the MWNT electrode [8, 31]. To obtain an optimized output energy from TECs, it is important to ensure that the temperature drop occur mainly between the electrodes, rather than across the bodies of two electrodes [8]. In order to eliminate the temperature loss, one of the most effective methods is to minimize the thermal resistance of the working electrodes.

In our experiment, the thermal resistance at the MWNT film/substrate junction was 0.0952 cm^2^ K W^−1^ under the substrate thickness of 500 μm measured by the transient hot wire method. Such a relative high thermal resistance weakens the performance improvement comparing with CVD growth of MWNT film, as shown in SI-2 of the ESI. Further efforts on the reduction of substrate thickness and improvement of the CNT purity are expected to enhance the thermal conductivity of the junction.

Normally, it is difficult to produce large area CNT electrode by CVD method due to growth non-uniformity and facility limitation. EPD technique is able to produce CNT films with large dimensions from simple setup. In this study, different sizes of MWNT electrodes were prepared by EPD to conduct the performance investigation. As shown in Fig. 4d, the lager the electrode area, the higher the output current and power, benefited from the increase of reaction sites [8]. When the electrode area was increased to 16 cm^2^, the output current and power could reach 7.0 mA and 166 μW, respectively, at the temperature difference of 40 °C. However, the increasing rates of *I*_sc_ and *P*_max_, where *P*_max_ was obtained by 1/4*V*_oc_ × *I*_sc_, declined gradually with the increase of surface area. Two factors, i.e., the edge effect of the CNT film [37] and the drop of MWNT density, may cause this nonlinear relation. Interestingly, the Seebeck coefficient increased from 1.42 to 2.40 mV K^−1^ with raising the surface area from 1 to 16 cm^2^, probably due to the concentration effect of the cell [6]. In our test system (hot-above-cold, see Fig. 4a), the buildup concentration would become obvious with increasing the surface area of the MWNT electrode, exhibiting partly the properties of the concentration cell [6]. Therefore, further efforts should be conducted to improve the TECs performances with large electrode area, e.g., trying to overcome the concentration effect with designs such as the flowing TECs [8, 38] or the cold-above-hot TECs [9]. The relation between the output power and the voltage is shown in the ESI as SI-1.

**Fig. 5:**
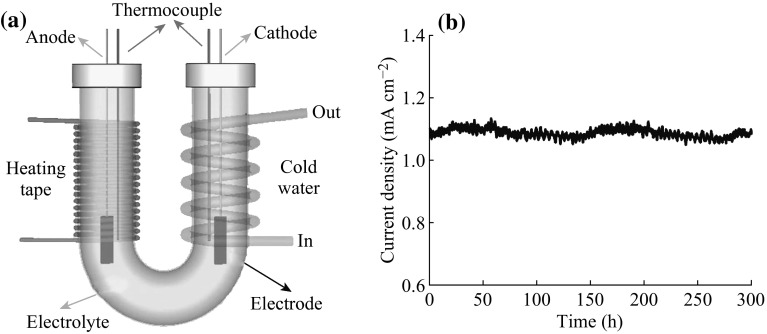
**a** The U-Shaped cell for long-term stability test. The distance between two electrodes was 7 cm and temperature difference was 15 °C. Cold side temperature was controlled by a recirculation water chiller under *T*_cold_ of 25 °C, and the hot side temperature was controlled by a resistive heater under *T*_hot_ of 40 °C. **b** *J*_SC_ stability over 300 h

If TECs were operated in an open system over a long period of time, the potassium ferrocyanide/ferricyanide solution would evaporate gradually, resulting in the current instability from the change of the electrolyte concentration. The TECs of closed system could operate with enhanced long-term stability [8]. In this work, the U-Shaped close system TECs with MWNT electrodes were employed to evaluate the long-term performance (see Fig. 5a). As shown in Fig. 5b, the U-shaped TEC discharged continuously for 300 h with no current degradation. The excellent discharge stability is benefited mainly from two aspects. At first, because the electrolyte in this system could reach equilibrium quickly, the concentration gradient from the mass transport kept almost unchanged during the operation [8, 31]. Secondly, the ESA of the MWNT film was stable during the operation of TECs due to the structure stability, showing strong adhesion between the MWNT film and the SS substrate. The excellent discharge property revealed great potential of the EPD production of CNT film for practical TEC electrodes.

## Conclusions

EPD is an efficient technique to produce MWNT electrodes for TECs applications. The relative power conversion efficiency of the MWNT TECs is 50 % higher than that of the platinum electrode-based TECs, while the excellent long-term current stability revealed the durability of the MWNT film. Furthermore, the low cost and large-scale production capabilities may promote the energy harvesting in various fields.

## Electronic supplementary material

Below is the link to the electronic supplementary material.

Supplementary material 1 (PDF 237 kb)
