# Supplementary material for: Thermo-Electrochemical Cells Based on Carbon Nanotube Electrodes by Electrophoretic Deposition
Source: Nanomicro Lett. 2016 Jan 29;8(3):240–6. doi: 10.1007/s40820-016-0082-8 (PMC6223680; doi:10.1007/s40820-016-0082-8)
Supplement: Supplementary file 1 — Supplementary material 1 (PDF 237 kb) [file 40820_2016_82_MOESM1_ESM.pdf]

Supplementary Information for

Thermo-Electrochemical Cells Based on Carbon Nanotube Electrodes by Electrophoretic Deposition

Weijin Qian[+], Mingxuan Cao[+], Fei Xie, Changkun Dong*

Institute of Mirco-Nano Structure & Optoelectronics, Wenzhou University, Wenzhou 325035, People's Republic of China

[+]Two authors contributed equally to this work

*Corresponding author. E-mail: dck@wzu.edu.cn

Tel: +86-577-86689067, Fax: +86-577-86689011

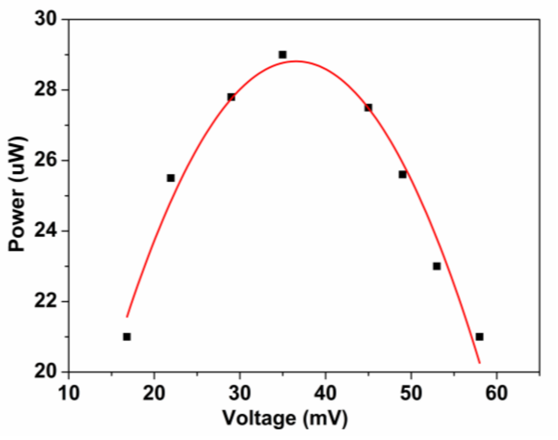

Fig. S1 Power versus Voltage between the two test electrodes. The distance between electrodes was 5 cm, the temperature difference was 50 ℃.

Table S1 Comparison of the performances of TECs based on MWNTs electrodes

Electrodes	j_{sc} (A m^{-2})	P_{max} (W m^{-2})	η_r (%)	References
MWNTs	45.2	0.82	0.9	this paper
MWNTs	85	1.8	1.4	Ref. 1

Note: The TEC performances from this work were not better than the results reported by Hu et al., mainly due to the higher thermal resistance

Reference

[1] R.C. Hu, B.A. Cola, N. Haram, J.N. Barisci, S. Lee et al., harvesting waste thermal energy using a carbon-nanotube-based thermo-electrochemical cell. Nano Lett. **10**(3), 838-846 (2010). doi:10.1021/nl903267n
